# Clinical characteristics and outcomes during a severe influenza season in China during 2017–2018

**DOI:** 10.1186/s12879-019-4181-2

**Published:** 2019-07-29

**Authors:** Xiaofang Fu, Yuqing Zhou, Jie Wu, Xiaoxiao Liu, Cheng Ding, Chenyang Huang, Shufa Zheng, Dhanasekaran Vijaykrishna, Yu Chen, Lanjuan Li, Shigui Yang

**Affiliations:** 1 State Key Laboratory for Diagnosis and Treatment of Infectious Diseases, Collaborative Innovation Center for Diagnosis and Treatment of Infectious Diseases, The First Affiliated Hospital, College of Medicine, Zhejiang University, Hangzhou, 310003 China; 2 Biomedicine Discovery Institute & Department of Microbiology, Monash University, Melbourne, VIC 3800 Australia

**Keywords:** The 2017–2018 influenza, Subtype, In-hospital fatality rates, Clinical characteristics, Antiviral therapy

## Abstract

**Background:**

A severe seasonal influenza epidemic was observed during 2017–2018 in China, prompting questions on clinical characteristics and outcomes of severe cases with influenza.

**Methods:**

We retrospectively collected clinical data and outcomes of laboratory-confirmed hospitalized patients (severe to critical) during Jan-2011 to Feb-2018 from five hospitals, followed by a systematic analysis of cases from 2017 to 2018 (*n* = 289) and all previous epidemics during 2011–2017 (*n* = 169).

**Results:**

In-hospital fatality was over 5-folds higher during the 2017–2018 (*p* < 0.01) in which 19 patients died (6.6%), whereas only 2 mortalities (1.2%) were observed during 2011–2017. Of the 289 hospitalized in 2017–2018, 153 were confirmed with influenza B virus, 110 with A/H1N1pdm09, and 26 A/H3N2, whereas A/H1N1pdm09 was the predominant cause of hospitalization in previous seasons combined (45%). Fatal cases in 2017–2018 were exclusively associated with either influenza B or A/H1N1pdm09. Our results show that a significant lower proportion of patients aged 14 or greater were treated with oseltamivir, during the 2017–2018 epidemic, and exhibited higher levels of clinical severity.

**Conclusions:**

In-hospital fatality rate might be significantly higher in the 2017–2018 season in China. A sufficient supply of oseltamivir and antiviral therapy within 48 h from onset could reduce fatality rates.

**Electronic supplementary material:**

The online version of this article (10.1186/s12879-019-4181-2) contains supplementary material, which is available to authorized users.

## Background

About 290,000 to 650,000 mortalities globally each year are linked to influenza [1] combined with a huge economic impact including both direct and indirect costs [2–4]. Following the influenza A (H1N1) pandemic in 2009–2010, there has been relatively low seasonal influenza activity in China until the nationwide epidemic during September 2017 and February 2018 resulting in increased influenza-related hospitalizations, severe illness and death [5]. This severe winter epidemic was reported to be predominated by the influenza B Yamagata lineage viruses and lower level circulation of A/H1N1 and H3N2 [5], however the recommended influenza vaccines for the 2017–2018 in China did not contain the Yamagata strain of influenza B [5].

Although clinical manifestations due to influenza A subtypes and B virus lineages are similar [6, 7], the virus type in circulation can affect the risk of infection among different age groups [8–10]. Owing to the frequent emergence of antigenic variants H3N2 predominant seasons are pronounced in all age groups [8], although variability in historic exposure to influenza antigenic variants over time will likely have an effect in the demographic distribution of influenza infection [11]. Data regarding possible differences in the clinical presentations and demography of hospitalization between seasons is not available [12].

Understanding the determinants of severe disease due to seasonal influenza infection is important for both the identification and effective management of high-risk cases and to infer whether the yearly variation in influenza lineage affects risk factors for severe disease – having implications beyond clinical management and public health policy [13]. Through a retrospective analysis of clinical and demographic characteristics of hospitalized influenza A and B cases from the five alliance hospitals in Zhejiang province, China during 2011–2018, this study aims to provide a scientific basis for the identification of high-risk cases associated with influenza.

## Methods

### Data source

**Fig. 1:**
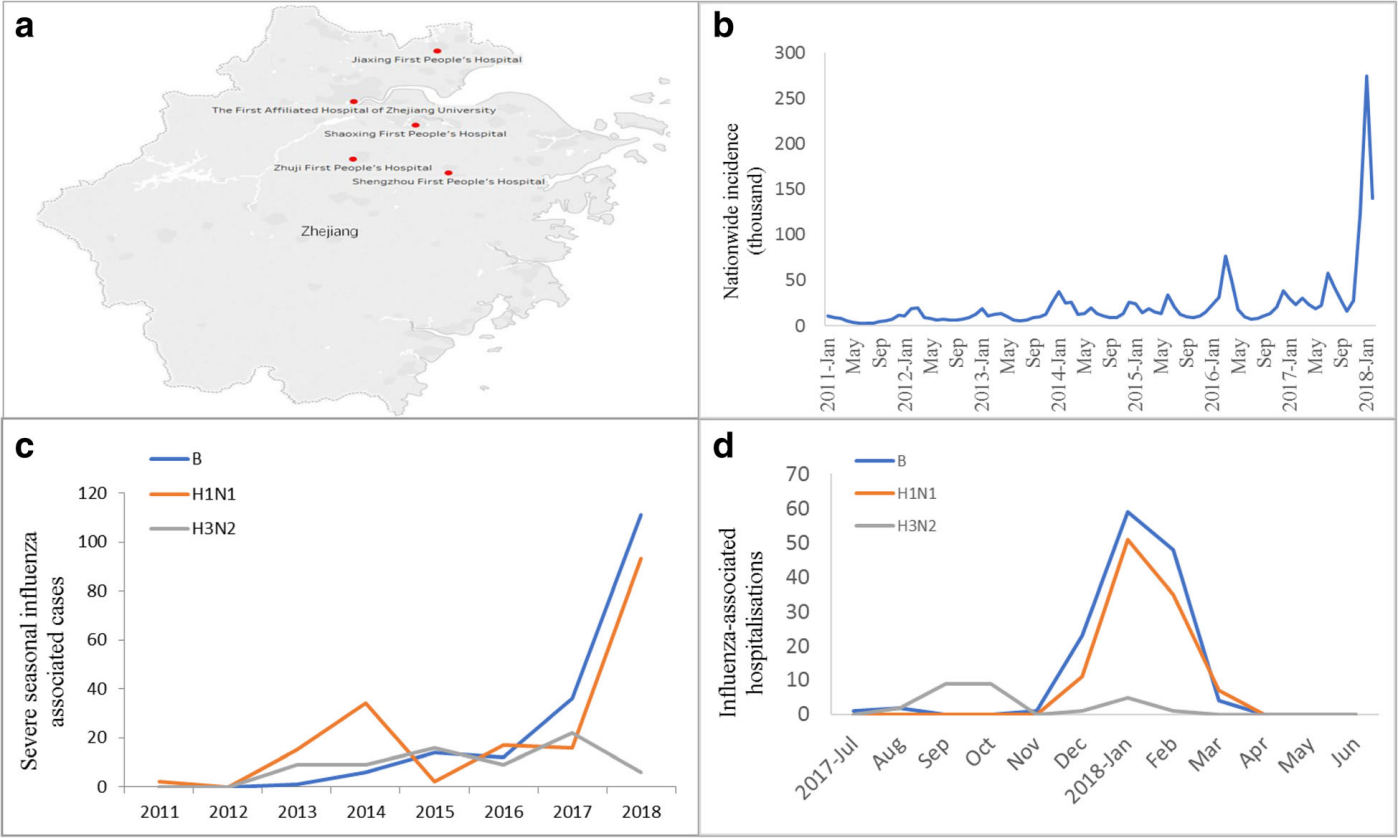
Surveillance sites and spatial-temporal distribution of influenza investigated in comparison to nationwide incidence. **a** Map showing the five alliance hospitals and their catchment area in Zhejiang. **b** Nationwide incidence of influenza; (**c**) Distribution of hospitalized cases by influenza subtypes by year from 2011 to 2018; (**d**) Distribution of hospitalized cases by influenza subtypes by month from July 2017 to June 2018

Clinical data of patients hospitalized with influenza during January 2011 and February 2018 were retrospectively collected from the five alliance hospitals in Zhejiang province, China (Fig. 1a). The types of assays for influenza were conducted according the diagnostic criteria for influenza. A standard data collection form was used to record clinical information systematically from admission to discharge for each patient. A trained team of physicians and medical students reviewed the patient charts and recorded demographic, clinical, and laboratory information, chest X-ray, results of diagnostic testing for influenza, antiviral and corticosteroid treatment, non-invasive or invasive ventilation, clinical complications and outcome. We collected the blood biochemistry values within 24 h of admission and chest X-ray examination before admission or within 24 h of admission.

### Diagnostic criteria

Cases admitted during Jan 2011 to Feb 2018 [14] and were laboratory-confirmed with influenza A or B virus by real-time RT-PCR were recruited for this study, however viruses for which the specific subtype was not known was excluded. A severe case met at least one of the following criteria on admission: [1] high fever with ≥ 39 °C for > 3 days, with serious coughing, purulent sputum, blood sputum or chest pain; [2] ≥ 20/min respiratory rate with dyspnea or cyanosis; [3] mental symptoms including slow reaction, drowsiness, restlessness or convulsions; [4] serious vomiting, diarrhea with dehydration; [6] new radiographic abnormality indicating pneumonia (on chest radiograph or CT scan); and [7] deterioration of underlying diseases [14]. A critical case met at least one of the following criteria on admission: (1) respiratory failure; (2) acute necrotic encephalopathy; (3) septic shock; (4) multiple organs insufficiency; and (5) other critical clinical conditions requiring intensive care. Outpatients or < 2 h hospitalization in emergency rooms, or with incomplete record of clinical outcome were excluded from our study [14]. Detailed clinical information along with laboratory criteria for recorded co-morbidities (See Additional file 1: Material 1) and specific reference ranges used to define abnormalities in blood results are provided in supplementary information (See Additional file 2: Table S1).

### Statistical analysis

The main outcome was all-cause mortality that occurred during a given hospital stay. Means (standard deviations) or medians (interquartile, IQR) were calculated as summaries of continuous variables. For categorical variables, percentages of patients in each category were calculated. We compared clinical characteristics and outcomes by chi-square test, Fisher’s exact test or non-parametric test, as appropriate. The adjusted odds ratios (OR) of the influenza season, and the corresponding 95% confidence intervals (CI) were calculated using multiple logistic regression models, adjusted for age and sex. *P* < 0.05 was considered statistically significant. The database was built with Excel software and IBM SPSS statistics (version 24) software was used for data extraction and description.

## Results

This retrospective study included 458 hospitalized cases with influenza A or B confirmed to subtype/lineage in five alliance hospitals in Zhejiang, China during 2011–2018 with accurate and detailed diagnosis, treatment, prognosis and demographic information, including 29 whose prognosis could be confirmed following a telephone follow-up. According to the reported epidemic onset during September 2017 [15, 16] we divided the cases into two groups, the 2017–2018 season and the previous one from the 2011–2017 seasons. 169 inpatients during the 2011–2017 seasons (77 cases of A/H1N1, 48 cases of A/H3N2, 44 cases of with influenza B) and 289 inpatients during the 2017–2018 season (110 cases of A/H1N1, 26 cases of A/H3N2, 153 cases of with influenza B). None of the patients in this study was found to be vaccinated with influenza vaccine before the onset.

The in-hospital fatality rate during the 2017–2018 epidemic was five-fold higher with 19 inpatient deaths (6.6%) (Table 1, Additional file 3: Figure S1), all of which were associated with either B (7.2%) or H1N1 (7.3%) (Table 2, Additional file 4: Figure S2) in contrast to two fatalities during all previous seasons combined (1.2%; A/H1N1 (1.3%) and A/H3N2 (2.1%). Fatality in over 60 years old (10.6%) was significantly higher than among the 14–59 years old population (4.7%) during 2017–2018, while no fatality was observed among children less than 14 in our cohort (Table 3). Respiratory failure (13.7% vs. 0.0%, *p* < 0.05) occurred more frequently in patients infected by influenza B virus during 2017–2018 (Additional file 4: Figure S2). Complication of those infected by the influenza A/H1N1 were significantly different between the 2011–2017 and 2017–2018 seasons (*p* < 0.01) but the prognosis was not significantly different (Additional file 4: Figure S2). There were no significant differences in the complication and prognosis due to A/H3N2 between the seasons (Additional file 4: Figure S2).

**Table 1:** Clinical characteristic of influenza associated hospitalizations during the 2017-2018 influenza and 2011-2017 seasons

Variables n (%)	2011-2017 (*n* = 169)	2017-2018 (*n* = 289)	*P* value
Gender-male	102 (60.4)	169 (58.5)	0.693
Age groups			
<14 years	22 (13.0)	37 (12.8)	
14- years	81 (47.9)	129 (44.6)	0.751
>=60 years	66 (39.1)	123 (42.6)	
Co-morbidities			
Cardiovascular diseases	55 (32.5)	120 (41.5)	0.056
Respiratory diseases	17 (5.9)	24 (8.3)	0.526
Chronic renal diseases	11 (6.5)	22 (7.6)	0.659
Chronic liver diseases	11 (6.5)	27 (9.3)	0.289
Diabetes mellitus	18 (10.7)	29 (10.0)	0.834
Cancer and hematological diseases	56 (33.1)	83 (28.7)	0.321
Stroke and Neuromuscular diseases	15 (8.9)	17 (5.9)	0.225
Immunosuppressant	19 (11.2)	38 (13.1)	0.551
Pregnancy	0 (0)	2 (0.7)	0.533
Postmortum within 30 days after delivery	0 (0)	1 (0.3)	1.000
Current smoking	**20 (6.9)**	57 (19.7)^*^	0.029
Received seasonal or influenza A (H1N1) vaccination	0 (0)	0 (0.0)	NA
Symptoms and Lab findings			
Fever (temp >=38)	159 (94.1)	257 (88.9)	0.065
Cough	141 (83.4)	247 (85.5)	0.559
Dyspnea	**17 (5.9)**	50 (17.3)^*^	0.034
Hemoptysis	5 (3.0)	14 (4.8)	0.329
CNS symptom	2 (1.2)	11 (3.8)	0.180
WBC<4	50 (29.6)	70 (24.2)	0.266
L%<20%	97 (59.1)	162 (56.1)	0.192
Platelet<100	36 (65.0)	46 (15.9)	0.286
ALT >40	**36 (21.4)**	86 (30.9)^*^	0.029
AST >40	54 (32.3)	109 (39.4)	0.137
LDH >300	48 (35.6)	108 (42.7)	0.172
CK>200	**14 (10.4)**	54 (21.3)^**^	0.007
CRP>8	121 (79.1)	196 (75.1)	0.355
ESR >20	67 (39.6)	98 (60.1)	0.420
Treatment on admission			
Antibiotics use	**160 (94.7)**	258 (89.3)^*^	0.048
	21 (12.4)	33 (11.4)	0.747
Oseltamivir	**147 (87.0)**	222 (76.8)^**^	0.008
Traditional Chinese Medicine	28 (16.6)	64 (22.1)	0.151
Glucocorticoids	64 (37.9)	134 (46.4)	0.077
Outcome			
Total In-hospital fatality	**2 (1.2)**	19 (6.6)^**^	0.008

*, **. Boldface values indicate (^*^P <.05; ^**^P <.01). Comparison of the 2017-2018 influenza season with control group (influenza season 2011-2017). WBC, white cell (×10^9^/L); L%, lymphocyte percent; PLT, platelet (×10^9^/L); *ALT* alanine aminotransferase (U/L), *AST* aspartate aminotransferase (U/L), *LDH* lactate dehydrogenase (U/L), *CK* creatine kinase (U/L), *CRP* C-reactive protein (mg/L), *ESR* erythrocyte sedimentation rate (mm/h). *IQR* interquartile range, *NA* not available

**Table 2:** Characteristics of inpatients with different subtypes of influenza viruses during the 2011–2017 and 2017–2018 influenza seasons

Variables n (%)	B		A/H1N1		A/H3N2	
	2011–2017 (*n* = 44)	2017–2018 (*n* = 153)	2011–2017 (*n* = 77)	2017–2018 (*n* = 110)	2011–2017 (*n* = 48)	2017–2018 (*n* = 26)
Gender-male	25 (56.8)	92 (60.1)	45 (58.4)	66 (60.0)	**32 (66.7)**	11 (42.3)^*^
Age groups						
< 14 years	**21 (47.7)**	**23 (15.0)**	**1 (1.3)**	**13 (11.8)**	0 (0)	1 (3.8)
14–59 years	**16 (36.4)**	**62 (40.5)**	**47 (61.0)**	57 (51.8)^*^	18 (37.5)	10 (38.5)
> =60 years	**7 (15.9)**	68 (44.4)^**^	**29 (37.7)**	**40 (36.4)**	30 (62.5)	15 (57.7)
Co-morbidities						
Cardiovascular diseases	**7 (15.9)**	55 (35.9)^*^	**50 (64.9)**	49 (44.5)^**^	21 (43.8)	16 (61.5)
Respiratory diseases	2 (4.5)	13 (8.5)	7 (9.1)	10 (9.1)	8 (16.7)	1 (3.8)
Chronic renal diseases	2 (4.5)	12 (7.8)	6 (7.8)	9 (8.2)	3 (4.5)	1 (3.8)
Chronic liver diseases	3 (6.8)	18 (11.8)	4 (5.2)	8 (7.3)	4 (8.3)	1 (3.8)
Diabetes mellitus	1 (2.3)	15 (9.8)	9 (11.7)	12 (10.9)	8 (16.7)	2 (7.7)
Cancer and hematological diseases	9 (20.5)	53 (34.6)	27 (35.1)	25 (22.7)	20 (41.7)	5 (19.2)
Stroke and Neuromuscular diseases	2 (4.5)	11 (7.2)	6 (7.8)	4 (3.6)	7 (14.6)	2 (7.7)
Immunosuppressant	**2 (4.5)**	27 (17.6)^*^	13 (16.9)	10 (9.1)	4 (8.3)	1 (3.8)
Pregnancy	0 (0)	0 (0)	0 (0)	2 (1.8)	0 (0)	0 (0)
Postmortum within 30 days after delivery	0 (0)	1 (0.7)	0 (0)	0 (0)	0 (0)	0 (0)
Current smoking	**3 (6.8)**	31 (20.3)^*^	13 (16.9)	21 (19.1)	4 (8.3)	5 (19.2)
Received seasonal or influenza A (H1N1) vaccination	0 (0)	0 (0)	0 (0)	0 (0)	0 (0)	0 (0)
Symptoms and Lab findings						
Fever (temp > = 38)	43 (97.7)	136 (88.9)	71 (92.2)	98 (89.1)	45 (93.8)	23 (88.5)
Cough	32 (72.7)	125 (81.7)	70 (90.9)	97 (88.2)	39 (81.3)	25 (96.2)
Dyspnea	3 (6.8)	13 (8.5)	**9 (11.7)**	31 (28.2)^**^	5 (10.4)	6 (23.1)
Hemoptysis	0 (0)	6 (3.9)	4 (5.2)	8 (7.3)	1 (2.1)	0 (0)
CNS symptom	0 (0)	2 (1.3)	1 (4.3)	8 (7.3)	1 (2.1)	1 (3.8)
WBC < 4	13 (29.5)	40 (26.1)	**26 (33.8)**	19 (17.3)^**^	11 (22.9)	11 (42.3)
L% < 20%	17 (40.5)	81 (53.3)	49 (63.6)	69 (63.3)	**31 (68.9)**	12 (46.2)^*^
Platelet< 100	7 (15.9)	35 (22.9)	**17 (22.1)**	10 (9.1)^*^	12 (25.0)	1 (3.8)
ALT > 40	8 (18.2)	38 (25.9)	**18 (23.7)**	45 (42.5)^**^	10 (20.8)	3 (12.0)
AST > 40	21 (48.8)	49 (33.1)	**22 (28.9)**	54 (51.4)^**^	11 (22.9)	6 (25.0)
LDH > 300	**21 (55.3)**	45 (35.7)^*^	**19 (33.3)**	61 (58.7)^**^	8 (20.0)	2 (8.7)
CK > 200	5 (13.5)	18 (14.4)	**5 (8.8)**	30 (28.6)^**^	4 (10.0)	6 (26.1)
CRP > 8	22 (57.9)	96 (70.1)	60 (87.0)	79 (79.8)	39 (84.8)	21 (84.8)
ESR > 20	15 (60.0)	46 (59.0)	34 (69.4)	45 (64.3)	18 (62.1)	7 (46.7)
Treatment on admission						
Antibiotics use	39 (88.6)	133 (86.9)	74 (96.1)	101 (91.8)	47 (97.9)	24 (92.3)
Mechanical ventilation	2 (4.5)	7 (4.6)	15 (19.5)	22 (20.0)	4 (8.3)	4 (15.4)
Oseltamivir	27 (61.4)	109 (71.2)	**75 (97.4)**	88 (80.0)^**^	45 (93.8)	25 (96.2)
Traditional Chinese Medicine	6 (13.6)	35 (22.9)	**8 (10.4)**	28 (25.5)^*^	**14 (29.2)**	1 (3.8)^*^
Glucocorticoids	**9 (20.5)**	65 (42.5)^**^	35 (45.5)	63 (57.3)	20 (41.7)	6 (23.1)
Outcome						
In-hospital fatality	0 (0)	11 (7.2)	1 (1.3)	8 (7.3)	1 (2.1)	0 (0)

*WBC* white cell (× 10^9^/L), *L* lymphocyte percent, *PLT* platelet (× 10^9^/L), *ALT* alanine aminotransferase (U/L), *AST* aspartate aminotransferase (U/L), *LDH* lactate dehydrogenase (U/L), *CK* creatine kinase (U/L), *CRP* C-reactive protein (mg/L), *ESR* erythrocyte sedimentation rate (mm/h), *IQR* interquartile range

*, ** Boldface values indicate (*P < .05; **P < .01). Comparison of influenza season 2017–2018 with control group (influenza season 2011–2017) among same type and subtypes of seasonal influenza viruses

**Table 3:** Characteristics of inpatients with influenza among different age groups during the 2011–2017 and 2017–2018 influenza seasons

Variables n (%)	< 14 years		14–59 years		> = 60 years	
	2011–2017 (*n* = 22)	2017–2018 (*n* = 37)	2011–2017 (*n* = 81)	2017–2018 (*n* = 129)	2011–2017 (*n* = 66)	2017–2018 (*n* = 123)
Gender-male	12 (54.5)	16 (43.2)	41 (50.6)	71 (55.0)	49 (74.2)	82 (66.7)
Co-morbidities						
Cardiovascular diseases	1 (4.5)	1 (2.7)	17 (21.0)	37 (28.7)	37 (56.1)	82 (66.7)
Respiratory diseases	0 (0)	0 (0)	3 (3.7)	2 (1.6)	14 (21.2)	22 (17.9)
Chronic renal diseases	0 (0)	0 (0)	5 (6.2)	11 (8.5)	6 (9.1)	11 (8.9)
Chronic liver diseases	0 (0)	0 (0)	8 (9.9)	14 (10.9)	3 (4.5)	13 (10.6)
Diabetes mellitus	0 (0)	0 (0)	5 (6.2)	9 (7.0)	13 (19.7)	20 (16.3)
Cancer and hematological diseases	3 (13.6)		28 (34.6)	39 (30.2)	25 (37.9)	37 (30.1)
Stroke and Neuromuscular diseases	0 (0)	0 (0)	4 (4.9)	2 (1.6)	11 (16.7)	15 (12.2)
Immunosuppressant	0 (0)	0 (0)	13 (16.5)	21 (16.3)	6 (9.1)	17 (13.8)
Pregnancy	0 (0)	0 (0)	0 (0)	2 (1.6)	0 (0)	0 (0)
Postmortum within 30 days after delivery	0 (0)	0 (0)	0 (0)	1 (0.8)	0 (0)	0 (0)
Current smoking	0 (0)	0 (0)	**7 (8.6)**	26 (20.2)^*^	13 (19.7)	31 (25.2)
Received seasonal or influenza A (H1N1) vaccination	0 (0)	0 (0)	0 (0)	0 (0)	0 (0)	0 (0)
Symptoms and Lab findings						
Fever (temp > = 38)	21 (95.5)	37 (100.0)	79 (97.5)	120 (93.0)	59 (89.4)	100 (81.3)
Cough	17 (77.3)	32 (86.5)	64 (79.0)	107 (82.9)	60 (90.1)	108 (87.8)
Dyspnea	0 (0)	3 (8.1)	6 (7.4)	20 (15.5)	11 (16.7)	27 (22.0)
Hemoptysis	0 (0)	0 (0)	3 (3.7)	9 (7.0)	2 (3.0)	5 (4.1)
CNS symptom	0 (0)	0 (0)	1 (1.2)	4 (3.1)	1 (1.5)	7 (5.7)
WBC < 4 (× 10^9^/L)	5 (22.7)	8 (21.6)	29 (35.8)	40 (31.0)	16 (24.2)	22 (17.9)
L% < 20%	4 (18.2)	9 (25.7)	51 (63.0)	67 (51.9)	42 (63.6)	86 (69.9)
Platelet< 100 (× 10^9^/L)	1 (4.5)	2 (5.4)	18 (22.2)	22 (17.1)	17 (25.8)	22 (17.9)
ALT > 40	1 (4.5)	4 (11.1)	20 (24.7)	48 (38.1)	15 (22.7)	34 (29.3)
AST > 40	12 (57.1)	22 (64.7)	24 (30.0)	50 (40.0)	18 (27.3)	37 (31.4)
LDH > 300	15 (75.0)	25 (73.5)	19 (33.9)	43 (38.4)	14 (23.7)	40 (37.4)
CK > 200	4 (21.1)	10 (29.4)	**4 (7.1)**	26 (23.0)^*^	6 (10.2)	18 (17.0)
CRP > 8	5 (31.3)	10 (32.3)	60 (81.1)	95 (79.8)	56 (88.9)	91 (82.0)
ESR > 20	2 (20.0)	4 (25.0)	30 (57.7)	46 (59.7)	**35 (85.4)**	48 (68.6)^*^
Treatment on admission						
Antibiotics use	19 (86.4)	30 (81.1)	76 (93.8)	118 (91.5)	**65 (98.5)**	110 (89.4)^*^
Mechanical ventilation	1 (4.5)	0 (0)	9 (11.1)	13 (10.1)	11 (16.7)	20 (16.3)
Oseltamivir	8 (36.4)	27 (73.0)	**76 (93.8)**	99 (76.7)^**^	**63 (95.5)**	96 (78.0)^**^
Traditional Chinese Medicine	2 (9.1)	6 (16.2)	15 (18.5)	38 (29.5)	11 (16.7)	20 (16.3)
Glucocorticoids	2 (9.1)	7 (18.9)	37 (45.7)	65 (50.4)	25 (37.9)	62 (50.4)
Outcome						
In-hospital fatality	0 (0)	0 (0)	1 (1.2)	6 (4.7)	**1 (1.5)**	13 (10.6)^*^

*WBC* white cell (× 10^9^/L), *L* lymphocyte percent, *PLT* platelet (× 10^9^/L), *ALT* alanine aminotransferase (U/L), *AST* aspartate aminotransferase (U/L), *LDH* lactate dehydrogenase (U/L), *CK* creatine kinase (U/L), *CRP* C-reactive protein (mg/L), *ESR* erythrocyte sedimentation rate (mm/h), *IQR* interquartile range

*, ** Boldface values indicate (*P < .05; **P < .01). Comparison of influenza season 2017–2018 with control group (influenza season 2011–2017) among same age groups

Inpatients with influenza during 2017–2018 smoked more frequently (19.7% vs. 6.9%, *p* < 0.05) (Table 1), especially among the 14–59 years category (Table 3), and experienced shortness of breath (dyspnea) more frequently (17.3% vs. 5.9%, *p* < 0.05), and were significantly higher in alanine aminotransferase (ALT) (30.9% vs. 21.4%, *p* < 0.05) and creatine kinase (CK) (21.3% vs. 10.4%, *p* < 0.01). However, fewer of the 2017–2018 patients were treated with antibiotics (89.3% vs. 94.7%, *p* < 0.05) (Table 1). Though of the improved coverage rate of oseltamivir for younger patients from 36.4% in 2011–17 seasons to 73.0% in 2017/18 season, owing to the shortages of oseltamivir, the total number of patients treated with oseltamivir prior to 48 h from onset in 2017/18 season were significantly lower than that in 2011–17 seasons (76.8% vs. 87.0%, *p* < 0.01), especially in the 14–59 (76.7% vs. 93.8%, *p* < 0.05) and > 60 (78.0% vs. 95.5%, *p* < 0.01) groups.

Fatality was higher in patients who did not receive antiviral therapy in 48 h from onset (B virus, 6.1%, and H1N1, 5.9%, respectively), than those who received antiviral therapy in 48 h from onset (2.9% and 0, respectively), although no significance was found. Elevated ALT was more common among those infected by influenza B who received antiviral therapy in 48 h from onset (*p* < 0.05). Those infected by the influenza A/H1N1 who did not receive antiviral therapy in 48 h from onset were more frequently experiencing cough (94.1% vs. 67.6%, *p* < 0.01), leukopenia (25.5% vs. 17.6%, *p* < 0.05) and increasing in CRP (85.7% vs. 67.9%, *p* < 0.05), LDH (54.4% vs. 24.0%, *p* < 0.01) and ESR (71.2% vs. 33.3%, *p* < 0.01) (Additional file 5: Table S2).

Among influenza B infection, compared to that in 2011–2017 seasons, hospitalized patients aged > 60 (*p* < 0.01), cardiovascular diseases (35.9% vs. 15.7%, *p* < 0.05), and use immunosuppressant (17.6% vs. 4.5%, *p* < 0.05) and smoking (20.3% vs. 6.8%, *p* < 0.05) were more common, and elevated lactate dehydrogenase (LDH) (35.7% vs. 55.3%, *p* < 0.05) were fewer in 2017–2018 seasons. Among influenza A/H1N1 infection, compared to that in 2011–2017 seasons, patients aged 14–59 years (*p* < 0.05), experienced dyspnea (28.2% vs. 11.7%, *p* < 0.05), elevated ALT (42.5% vs. 23.7%, *p* < 0.01), aspartate aminotransferase (AST) (51.4% vs. 28.9%, *p* < 0.01), LDH (58.7% vs. 33.3%, *p* < 0.01) and CK (28.6% vs. 8.8%, *p* < 0.01) were more common. While those infected by the influenza A/H1N1 during the 2017–2018 season experienced leukopenia (17.3% vs. 33.8%, *p* < 0.01) and thrombocytopenia (9.1% vs. 22.1%, *p* < 0.05) less frequently and used less oseltamivir (80.0% vs. 97.4%, *p* < 0.01). Fewer males were infected with A/H3N2 during the 2011–2017 seasons (42.3% vs. 66.7%, *p* < 0.05), and were less likely to experience lymphopenia (46.2% vs. 68.9%, *p* < 0.05). (Table 2).

Adjusted for age and gender, increased risk of death for 2017–2018 influenza were associated patients with co-morbidities of cancer and hematological diseases (adjusted OR [aOR], 3.1; 95%CI, 1.2–8.1), leukopenia (aOR, 3.5; 95%CI, 1.2–10.4), elevated LDH (aOR, 7.6; 95%CI, 2.3–25.2), elevated AKI (aOR, 10.1; 95%CI, 2.9–34.6), DIC (aOR, 56.4; 95%CI, 14.5–219.3), secondary bacterial infection (aOR, 4.4; 95%CI, 1.6–12.4); ARDS (aOR, 32.2; 95%CI, 8.7–112.2), respiratory failure (aOR, 11.7; 95%CI, 4.0–34.2), shock (aOR, 23.7; 95%CI, 4.9–115.2) and multiple organ failure (MOF) (aOR, 15.5; 95%CI, 3.0–79.5). Glucocorticoids treatments (aOR, 11.6; 95%CI, 2.6–52.4) during the 2017–2018 season was also associated with increased risk of death (Table 4). However, the increased risk factors of death for 2017–2018 season influenza were not found to be statistical significance for 2011–2017 seasons influenza. Poisson regression for predicting death of patients further indicated that 2017–2018 influenza seasons increased the risk of death (HR, 1.1; 95% CI, 0.4–1.9), DIC and ARDS are significant predictors, with HRs of 2.2 (95%CI, 1.1–3.2) and 1.4 (95%CI, 0.3–2.6), respectively (Additional file 6: Table S3).

**Table 4:** Age- and gender-adjusted-risk factors for death of patients during the 2011–2017 and 2017–2018 influenza seasons

Risk factors	2017–2018		2011–2017	
	n (%)	Adjusted OR(95%CI)	n (%)	Adjusted OR(95%CI)
Cancer and Hematological diseases	83 (28.7)	3.1 (1.2–8.1)	56 (33.1)	0.0 (0.0-)
WBC < 4	70 (24.2)	3.5 (1.2–10.4)	50 (29.6)	2.3 (0.0-)
LDH > 300	108 (42.7)	7.6 (2.3–25.2)	48 (35.6)	1.0 (0.0-)
AKI	19 (6.6)	10.1 (2.9–34.6)	4 (2.4)	0.0 (0.0-)
DIC	20 (6.9)	56.4 (14.5–219.3)	3 (1.8)	1.9 (0.0-)
Secondary bacterial infection	72 (24.9)	4.4 (1.6–12.4)	13 (7.7)	2.8 (0.0-)
ARDS	20 (6.9)	32.2 (8.7–112.2)	2 (1.2)	NA
Respiratory Failure	62 (21.5)	11.7 (4.0–34.2)	10 (5.9)	3.2 (0.0-)
Shock	9 (3.1)	23.7 (4.9–115.2)	1 (0.6)	0.0 (0.0-)
MOF	9 (3.1)	15.5 (3.0–79.5)	0 (0)	NA
Glucocorticoids	134 (46.4)	11.6 (2.6–52.4)	64 (37.9)	1.5 (0.1–25.5)

*AKI* acute kidney injury, *DIC* disseminated intravascular coagulation, *ARDS* acute respiratory distress syndrome, *MOF* multiple organ failure

*WBC*, white cell (10^9^/L), *LDH* lactate dehydrogenase (u/L)

*CI* confidence interval, *OR* odds ratio, *NA* not available

## Discussions

Our study shows that there was a five-fold higher rate of the in-hospital fatality rate during the 2017–2018 winter season in China, a season characterized by high levels of outpatient clinic and emergency department visits for influenza-like illness (ILI) [17]. In-hospital fatality rate during 2017–2018 was significantly higher in over 60 years than in previous seasons, with a predominant number of infections due to influenza B and A/H1N1 compared with A/H3N2. Hospitalizations during the 2017–2018 epidemic were predominantly due to B viruses as indicated by early nationwide surveillance reports [5]. A predominant number of H3N2 hospitalizations occurred during late summer and autumn months of August–October 2017 prior to the observation of H1N1 and B cases, coinciding with reports of the H3N2 summer epidemic in southern China [18] where seasonality is bimodal [19]. The significantly larger number of influenza hospitalized cases from 2017 to 2018 might be attributed to higher detection rate and more frequent turnover time of beds. A significantly larger number of hospitalized patients during 2017–2018 did not receive antiviral therapy within 48 h from symptom onset, and a higher number of these patients died, In addition, we found that inpatients with influenza during the 2017–2018 season were more frequently smoking than the 2011–2017 seasons, recognizing the increased risk of smoking during influenza epidemics and pandemics [20]. Our study highlights the importance of encouraging people to vaccinate against influenza, which remain a huge challenge in China– none of the inpatients in our study had received seasonal influenza vaccination prior to the epidemic.

When we analyzed the characteristics of inpatients with influenza among different age groups during the 2011–2017 and 2017–2018 seasons, we found less frequent prescription of oseltamivir to inpatients aged 14–59 years and over 60 years during the 2017–2018 season, but the inpatients who were younger than 14 years had increased coverage with oseltamivir. Following the standardization of influenza treatment with oseltamivir, the clinical demand for oseltamivir increased greatly, and medical institutions, pharmacy terminals and families of drug buyers all stocked oseltamivir in the short term, resulting in insufficient supply in the short-term in China [21]. Oseltamivir is the preferred treatment for younger patients, owing to shortage of manpower in the pediatric departments and the emotions of parents, and this also contributed to the abuse of oseltamivir [22]. More data and rationale are needed to define whether the insufficient supply of oseltamivir in adults (> 14 years) may have increased risk for death during influenza outbreak in China. Stockpiling of Tamiflu® (oseltamivir) against pandemic threats has been initiated by several countries, with Britain for example pushing ahead with its plan of stockpiling Tamiflu at £49 m (€60 m; $80 m) to maintain its stockpile of antivirals in case of a flu pandemic [23].

Ninety percent of inpatients during 2017–2018 had received antibiotics, despite previous studies among healthy adults showing the lack of effect on prognosis, and there is no data to confirm that antibiotics can prevent influenza or related complications [24, 25]. Conducive to the rational use of antibiotics by clinicians [26, 27], rapid, specific and cost effective diagnosis of influenza type is required [28, 29].

During the 2017–2018 season, risk factors for death included infection with influenza B virus, secondary bacterial infection, ARDS, respiratory failure, shock, MOF, cancer and hematological diseases, elevated WBC, elevated LDH, AKI, DIC and glucocorticoids treatment, were found in the individuals who had cardiovascular diseases or using immunosuppressant. We also found that those infected by the influenza B during the 2017–2018 season were more likely than the 2011–2017 seasons to have cardiovascular diseases and use immunosuppressant. Some studies found that the unpredictability of influenza B lineage circulation were associated with substantial morbidity [2, 30].

Clinical knowledge of identified potential factors for mortality may aid in the management of influenza infection. Multivariate analysis adjusted for age and gender showed that the hospitalized patients with influenza during the 2017–2018 season had many potential risk factors for death but age and gender were not associated with an increased risk of death among inpatients during the 2011–2017 seasons. Age, gender, and underlying health conditions should be considered when planning influenza vaccination and treatment strategies [31]. Risk factors for death during the 2017–2018 season were mainly associated with complications: pulmonary complications were most common, but included secondary bacterial pneumonia in children and ARDS in adults [32]. Respiratory failure, ALI and secondary bacterial infection were common pulmonary complications during the 2017–2018 season.

A potential limitation of this study is that we may had overestimated the in-hospital fatality due to influenza, since inclusion criteria required an identification of specific influenza types and subtypes, and deaths were more likely to receive clinically clear diagnosis than survival cases. However, this limitation does not have an effect on the comparison of different influenza seasons. Because the number of influenza patients had increased significantly in 2017/18 season, and hospital beds are limited and stable, only more severe patients can be admitted to the hospital this season. We may had overestimated the in-hospital fatality in 2017/18 season due to the selection bias. Our study focused on infection due to types and subtypes of seasonal influenza viruses during the 2011–2018, however comparison to other respiratory infections such as the respiratory syncytial virus and adenovirus should be further explored to better understand pathogenesis due to influenza viruses. Additionally, due to the collection of data from five different hospitals, inherent differences in practice and training over time may have had a bias in case selection. Finally, we did not include cases of mixed infections (the mixed infection of influenza A/H1N1 and influenza B; the mixed infection of influenza A/H1N1 and A/H3N2) and cases of influenza A that were not specified classified, so data on these cases could have some impact on our results.

## Conclusions

In-hospital fatality rate might be significantly higher in the 2017–2018 season in China. Those who have some other chronic conditions, and those who had a few complications were more deaths in 2017–18 than in all other seasons combined. Disseminated intravascular coagulation and acute respiratory distress syndrome were significant predictors for die risk during 2017–2018 influenza seasons. A sufficient supply of oseltamivir and antiviral therapy in 48 h from onset could reduce fatality rates.

## Additional files

Additional file 1: **Material 1.** Detailed clinical information along with laboratory criteria for recorded co-morbidities. (PDF 56 kb)

Additional file 2: **Table S1.** Specific reference ranges used to define abnormalities in blood results. (PDF 46 kb)

Additional file 3: **Figure S1.** Comparison of complication and prognosis between the 2011–2017 and 2017–2018 influenza seasons. The orange bar indicated the rates of complication and prognosis in 2017–2018 season, and the blue bar indicated the rates of complication and prognosis in 2011–2017 seasons. The single star “*” noted *p* < 0.05, and the double stars “**” noted *p* < 0.01. ARDS, acute respiratory distress syndrome; AMCI, acute myocardial infarction; DIC, disseminated intravascular coagulation; MOF, multiple organ failure; ALI, acute lung injury; MOF, multiple organ failure. Patients in 2017–2018 also presented frequently with acute respiratory distress syndrome (ARDS) (6.9% vs. 1.2%, *p* < 0.05), disseminated intravascular coagulation (DIC) (6.9% vs. 1.8%, *p* < 0.05) and multiple organ failure (MOF) (3.1% vs. 0.0%, *p* < 0.05), and were more likely to have respiratory failure (21.5% vs. 5.9%, *p* < 0.01), acute lung injury (ALI) (24.9% vs. 11.2%, *p* < 0.01) and secondary bacterial infection (24.9% vs. 7.7%, *p* < 0.01), than in all previous seasons. (PDF 175 kb)

Additional file 4: **Figure S2.** Comparison of complication and prognosis by age groups and subtypes between the 2011–2017 and 2017–2018 influenza seasons. The orange bar indicated the rates of complication and prognosis in 2017–2018 season, and the blue bar indicated the rates of complication and prognosis in 2011–2017 seasons. The left part of the figure showed comparison of complication and prognosis by age groups between the 2011–2017 and 2017–2018 influenza seasons; the right part of the figure showed comparison of complication and prognosis by subtypes between the 2011–2017 and 2017–2018 influenza seasons. The single star “*” noted *p* < 0.05, and the double stars “**” noted *p* < 0.01. ARDS, acute respiratory distress syndrome; ALI, acute lung injury. (PDF 185 kb)

Additional file 5: **Table S2.** Antiviral therapy and outcomes of inpatients with different subtypes of influenza viruses during the 2011–2018 seasons. (PDF 64 kb)

Additional file 6: **Table S3.** Poisson regression for predicting death of patients during the 2011–2017 and 2017–2018 influenza seasons. (PDF 44 kb)

## Data Availability

The datasets used and/or analyzed during the current study are available from the corresponding author on reasonable request.

## Abbreviations

AKI: Acute kidney injury
ALI: Acute lung injury
ALT: Alanine aminotransferase
AMI: Acute myocardial infarction
ARDS: Acute respiratory distress syndrome
AST: Aspartate aminotransferase
CI: Confidence interval
CK: Creatine kinase
CRP: C-reactive protein
DIC: Disseminated intravascular coagulation
ESR: Erythrocyte sedimentation rate
IQR: Interquartile range
L%: Lymphocyte percent
LDH: Lactate dehydrogenase
MOF: Multiple organ failure
NA: Not available
OR: Odds ratio
PLT: Platelet
WBC: White cell

## References

[1] WHO (2017). Up to 650 000 people die of respiratory diseases linked to seasonal flu each year.

[2] Keech DM, Beardsworth P (2008). The impact of Influenza on working days lost. Pharmacoeconomics.

[3] Reed C, Chaves SS, Kirley PD (2015). Estimating Influenza disease burden from population-based surveillance data in the United States. PLoS One.

[4] Iuliano AD, Roguski KM, Chang HH (2018). Estimates of global seasonal influenza-associated respiratory mortality: a modelling study. Lancet.

[5] Prevention CCfDCa. Recent seasonal Influenza epidemiology in China and knowledge of. Prev Control. 2018; http://www.chinacdc.cn/jkzt/crb/bl/lxxgm/zstd/201801/t20180108_158017.html. Accessed 11 Nov 2018.

[6] Miller M, Miller M (2012). Myocardial injury and bacterial pneumonia contribute to the pathogenesis of fatal Influenza B virus infection. J Emerg Med J Infect Dis.

[7] Kyung-Wook H, Hee Jin C, Joon Young S, Yun NJ, Tae Un Y, Woo Joo K (2015). Clinical manifestations of influenza a and B in children and adults at a tertiary hospital in Korea during the 2011-2012 season. Jpn J Infect Dis.

[8] Olson DR, Heffernan RT, Marc P, Kevin K, Don W, Farzad M (2007). Monitoring the impact of influenza by age: emergency department fever and respiratory complaint surveillance in new York City. PLoS Med.

[9] Hossein K, Farrell GM, Kirsten SG, Raul R (2009). Differences in patient age distribution between influenza a subtypes. PLoS One.

[10] Worby CJ, Chaves SS, Wallinga J, Lipsitch M, Finelli L, Goldstein E (2015). On the relative role of different age groups in influenza epidemics. Epidemics.

[11] Fonville JM, Wilks SH, James SL (2014). Antibody landscapes after influenza virus infection or vaccination. Science (New York, NY).

[12] Esposito S, Molteni CG, Daleno C (2011). Clinical and socioeconomic impact of different types and subtypes of seasonal influenza viruses in children during influenza seasons 2007/2008 and 2008/2009. BMC Infect Dis.

[13] WHO (2017). Standard guidelines for the clinical management of severe influenza virus infections. Initial Guideline Development Group (GDG) Meeting.

[14] China NHCotPsRo. The Notification of the Prevention and Treatment of the Flu in 2018. 2018. http://www.nhc.gov.cn/cms-search/xxgk/getManuscriptXxgk.htm?id=5737c258bb0c4a0493cb4f65fcf11be1. Accessed 14 Dec 2018.

[15] Prevention CsCfDCa. Answers on the issues related to recent influenza outbreaks from the China's Center for Disease Control and Prevention. 2018. http://www.chinacdc.cn/jkzt/crb/bl/lxxgm/zstd/201801/t20180108_158017.html. Accessed 10 Dec 2018.

[16] WHO (2018). Influenza Laboratory Surveillance Information.

[17] Prevention CsCfDCa. Answers on the issues related to recent influenza outbreaks from the China's Center for Disease Control and Prevention. 2018. https://mp.weixin.qq.com/s/hSWkd7Dz6UyTd8iPxoYaaw. Accessed 15 Dec 2018.

[18] Prevention CCfDCa (2017). The influenza epidemic level in southern China has fallen back.

[19] Yue-Long S, Li-Qun F, Vlas SJ, De YG, Jan Hendrik R, Wu-Chun C (2010). Dual seasonal patterns for influenza, China. Emerg Infect Dis.

[20] Epstein MA, Sadina R, Alvin Nelson EA (2010). Is smoking a risk factor for influenza hospitalization and death?. J Infect Dis.

[21] Su yi. Disclosure of oseltamivir. Med Econ J. 2018; Sect. 001.

[22] Zhou M, Lei-Lei LI, Mao CM, Bi-Jie HU. Deep analysis of influenza epidemic in China in this year. Chin J Nosocomiol. 2018;28(4):631-5.

[23] Torjesen I (2014). Tamiflu purchase worth £49m will go ahead, government says. BMJ.

[24] Little P, Rumsby K, Kelly J (2005). Information leaflet and antibiotic prescribing strategies for acute lower respiratory tract infection: a randomized controlled trial. JAMA.

[25] Carrat F, Schwarzinger M, Housset B, Valleron AJ (2004). Antibiotic treatment for influenza does not affect resolution of illness, secondary visits or lost workdays. Eur J Epidemiol.

[26] Wong DM, Blumberg DA, Lowe LG (2006). Guidelines for the use of antibiotics in acute upper respiratory tract infections. Am Fam Physician.

[27] Zoorob R, Sidani MA, Fremont RD, Kihlberg C (2012). Antibiotic use in acute upper respiratory tract infections. Am Fam Physician.

[28] Boonsuk P, Payungporn S, Chieochansin T (2008). Detection of influenza virus types a and B and type a subtypes (H1, H3, and H5) by multiplex polymerase chain reaction. Tohoku J Exp Med.

[29] Suwannakarn K, Payungporn S, Chieochansin T (2008). Typing (a/B) and subtyping (H1/H3/H5) of influenza a viruses by multiplex real-time RT-PCR assays. J Virol Methods.

[30] Caini S, Huang QS, Ciblak MA (2015). Epidemiological and virological characteristics of influenza B: results of the global Influenza B study. Influenza Other Respir Viruses.

[31] Quandelacy TM, Viboud C, Charu V, Lipsitch M, Goldstein E (2014). Age- and sex-related risk factors for influenza-associated mortality in the United States between 1997-2007. Am J Epidemiol.

[32] Michael BR, Sarah DH (2010). Complications of seasonal and pandemic influenza. Crit Care Med.

