# Supplementary material for: Clinical characteristics and outcomes during a severe influenza season in China during 2017–2018
Source: BMC Infect Dis. 2019 Jul 29;19:668. doi: 10.1186/s12879-019-4181-2 (PMC6664535; doi:10.1186/s12879-019-4181-2)
Supplement: Supplementary file 1 — Material 1. Detailed clinical information along with laboratory criteria for recorded co-morbidities. (PDF 56 kb) [file 12879_2019_4181_MOESM1_ESM.pdf]

Additional Material 1: Detailed clinical information along with laboratory criteria for recorded co-morbidities

Cardiovascular diseases: refers to one or more of the following symptoms: coronary heart disease, chronic congestive heart failure, valvular disease and hypertension. Respiratory diseases: refers to one or more of the following symptoms: chronic obstructive pulmonary disease, active tuberculosis and old tuberculosis with respiratory insufficiency. Diabetes mellitus: refers to diabetes mellitus type 1 and diabetes mellitus type 2. Immunosuppressant: patients with HIV/AIDS, or patients who were prescribed immunosuppressant agents, or corticosteroids (equivalent to prednisone 15mg/d, 30 days). CNS system symptoms: central nervous system symptoms; refers to one or more of the following symptoms: insomnia, restlessness, hallucination, headache, dizziness and abnormal behavior. AKI, acute kidney injury: serum creatinine increased by 2-fold or GFR decreased >50%, or urine,0.5 ml/kg/h for at least 12 hours. ARDS, acute respiratory distress syndrome: lung injury of acute onset, PaO2/FiO2<=200, bilateral opacities on chest imaging (chest radiograph or CT) not explained by other lung pathology (e.g. effusion, lobar/lung collapse, or nodules).
