# Supplementary material for: Clinical characteristics and outcomes during a severe influenza season in China during 2017–2018
Source: BMC Infect Dis. 2019 Jul 29;19:668. doi: 10.1186/s12879-019-4181-2 (PMC6664535; doi:10.1186/s12879-019-4181-2)
Supplement: Supplementary file 2 — Table S1. Specific reference ranges used to define abnormalities in blood results. (PDF 46 kb) [file 12879_2019_4181_MOESM2_ESM.pdf]

Additional Table 1 Specific reference ranges used to define abnormalities in blood results

Measure	Units	Rang
Leukopenia	10^9 cells per liter	<4
Lymphopenia	_	<20%
Thrombocytopenia	10^9 cells per liter	<100
Elevated aspartate aminotransferase (ALT)	U per liter	>40
Elevated alanine aminotransferase (AST)	U per liter	>40
Elevated LDH	U per liter	>300
Elevated CK	U per liter	>200
Elevated CRP	mg/l	>8
Elevated ESR	mm/h	>20
