# Supplementary material for: Clinical characteristics and outcomes during a severe influenza season in China during 2017–2018
Source: BMC Infect Dis. 2019 Jul 29;19:668. doi: 10.1186/s12879-019-4181-2 (PMC6664535; doi:10.1186/s12879-019-4181-2)
Supplement: Supplementary file 3 — Figure S1. Comparison of complication and prognosis between the 2011–2017 and 2017–2018 influenza seasons. The orange bar indicated the rates of complication and prognosis in 2017–2018 season, and the blue bar indicated the rates of complication and prognosis in 2011–2017 seasons. The single star “*” noted p < 0.05, and the double stars “**” noted p < 0.01. ARDS, acute respiratory distress syndrome; AMCI, acute myocardial infarction; DIC, disseminated intravascular coagulation; MOF, multiple organ failure; ALI, acute lung injury; MOF, multiple organ failure. Patients in 2017–2018 also presented frequently with acute respiratory distress syndrome (ARDS) (6.9% vs. 1.2%, p < 0.05), disseminated intravascular coagulation (DIC) (6.9% vs. 1.8%, p < 0.05) and multiple organ failure (MOF) (3.1% vs. 0.0%, p < 0.05), and were more likely to have respiratory failure (21.5% vs. 5.9%, p < 0.01), acute lung injury (ALI) (24.9% vs. 11.2%, p < 0.01) and secondary bacterial infection (24.9% vs. 7.7%, p < 0.01), than in all previous seasons. (PDF 175 kb) [file 12879_2019_4181_MOESM3_ESM.pdf]

Additional Figure 1 Comparison of complication and prognosis between the 2011-2017 and 2017-2018 influenza seasons

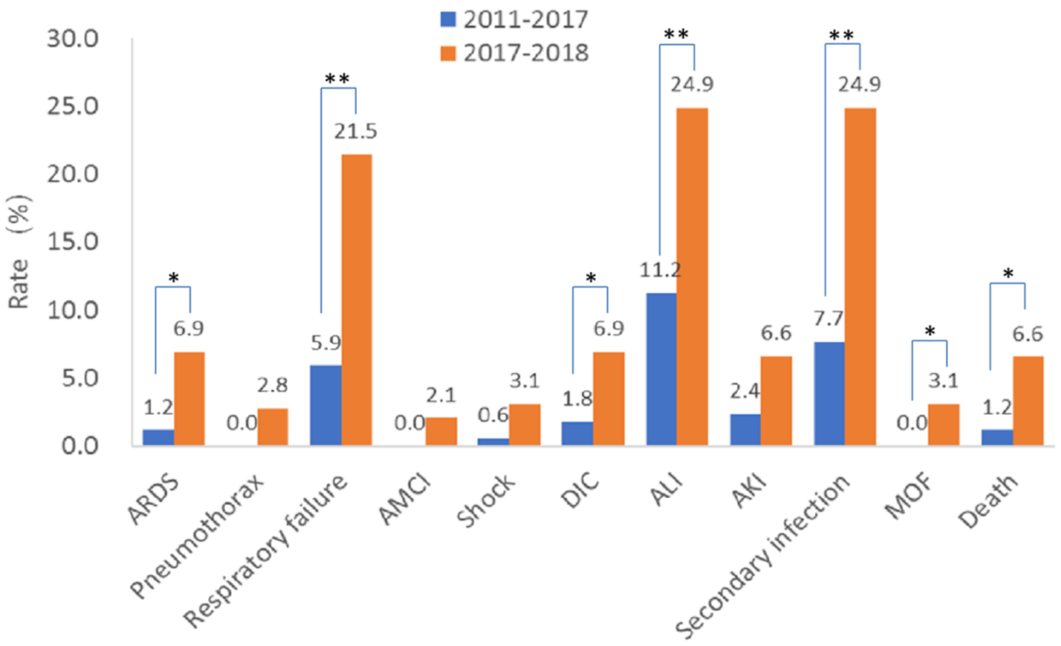

Figure legend: The orange bar indicated the rates of complication and prognosis in 2017-2018 season, and the blue bar indicated the rates of complication and prognosis in 2011-2017 seasons. The single star "*" noted p<0.05, and the double stars "**" noted p<0.01. ARDS, acute respiratory distress syndrome; AMCI, acute myocardial infarction; DIC, disseminated intravascular coagulation; MOF, multiple organ failure; ALI, acute lung injury; MOF, multiple organ failure.

Patients in 2017-2018 also presented frequently with acute respiratory distress syndrome (ARDS) (6.9% vs. 1.2%, p<0.05), disseminated intravascular coagulation (DIC) (6.9% vs. 1.8%, p<0.05) and multiple organ failure (MOF) (3.1% vs. 0.0%, p<0.05), and were more likely to have respiratory failure (21.5% vs. 5.9%, p<0.01), acute lung injury (ALI) (24.9% vs. 11.2%, p<0.01) and secondary bacterial infection (24.9% vs. 7.7%, p<0.01), than in all previous seasons.
