# Supplementary material for: Clinical characteristics and outcomes during a severe influenza season in China during 2017–2018
Source: BMC Infect Dis. 2019 Jul 29;19:668. doi: 10.1186/s12879-019-4181-2 (PMC6664535; doi:10.1186/s12879-019-4181-2)
Supplement: Supplementary file 4 — Figure S2. Comparison of complication and prognosis by age groups and subtypes between the 2011–2017 and 2017–2018 influenza seasons. The orange bar indicated the rates of complication and prognosis in 2017–2018 season, and the blue bar indicated the rates of complication and prognosis in 2011–2017 seasons. The left part of the figure showed comparison of complication and prognosis by age groups between the 2011–2017 and 2017–2018 influenza seasons; the right part of the figure showed comparison of complication and prognosis by subtypes between the 2011–2017 and 2017–2018 influenza seasons. The single star “*” noted p < 0.05, and the double stars “**” noted p < 0.01. ARDS, acute respiratory distress syndrome; ALI, acute lung injury. (PDF 185 kb) [file 12879_2019_4181_MOESM4_ESM.pdf]

**Additional Figure 2 Comparison of complication and prognosis by age groups and subtypes between the 2011-2017 and 2017-2018 influenza seasons**

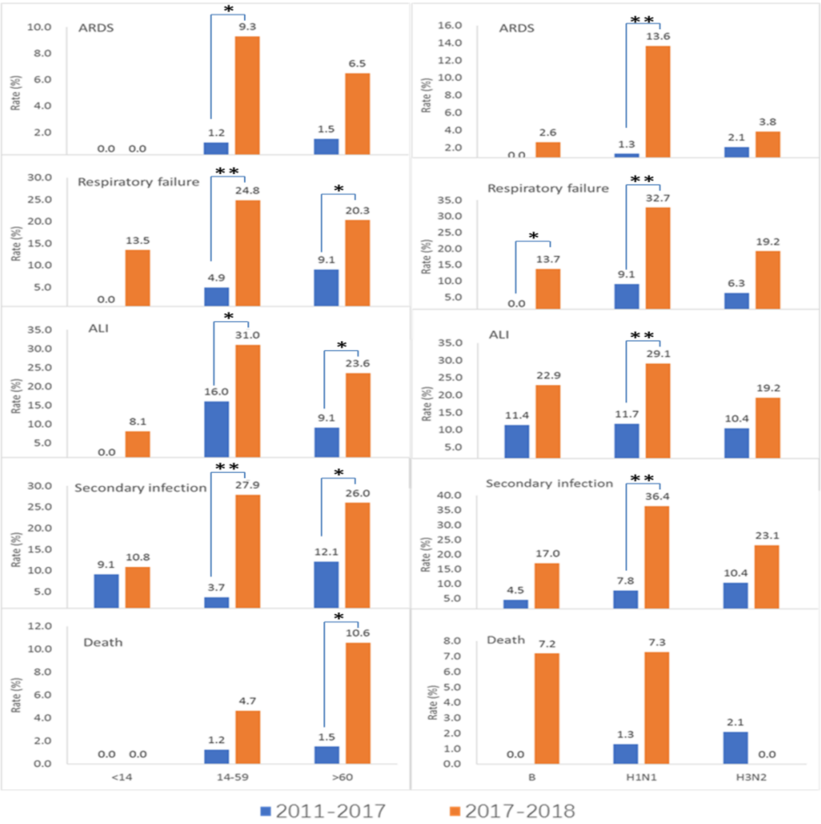


Figure legend: The orange bar indicated the rates of complication and prognosis in 2017-2018 season, and the blue bar indicated the rates of complication and prognosis in 2011-2017 seasons. The left part of the figure showed comparison of complication and prognosis by age groups between the 2011-2017 and 2017-2018 influenza seasons; the right part of the figure showed comparison of complication and prognosis by subtypes between the 2011-2017 and 2017-2018 influenza seasons. The single star "*" noted $p<0.05$, and the double stars "**" noted $p<0.01$. ARDS, acute respiratory distress syndrome; ALI, acute lung injury.
