# Supplementary material for: Clinical characteristics and outcomes during a severe influenza season in China during 2017–2018
Source: BMC Infect Dis. 2019 Jul 29;19:668. doi: 10.1186/s12879-019-4181-2 (PMC6664535; doi:10.1186/s12879-019-4181-2)
Supplement: Supplementary file 5 — Table S2. Antiviral therapy and outcomes of inpatients with different subtypes of influenza viruses during the 2011–2018 seasons. (PDF 64 kb) [file 12879_2019_4181_MOESM5_ESM.pdf]

Additional Table 2 Antiviral therapy and outcomes of inpatients with different subtypes of influenza viruses during the 2011-2018 seasons

Types and Subtypes	B		A/H1N1		A/H3N2	
Antiviral therapy in 48 hours from onset	Yes (n=34)	No (n=163)	Yes (n=34)	No (n=153)	Yes (n=19)	No (n=55)
Gender-male	25 (73.5)	92 (56.4)	20 (58.8)	91 (59.5)	10 (52.6)	33 (60.0)
Age groups						
<14 years	3 (8.8)	41 (25.2)	5 (14.7)	9 (5.9)	0 (0)	1 (1.8)
14-59 years	17 (50.0)	61 (37.4)	18 (52.9)	86 (56.2)	8 (42.1)	20 (36.4)
>=60 years	14 (41.2)	61 (37.4)	11 (32.4)	58 (37.9)	11 (57.9)	34 (61.8)
Co-morbidities						
Cardiovascular diseases	14 (41.2)	48 (29.4)	12 (35.3)	64 (41.8)	9 (47.4)	28 (50.9)
Respiratory diseases	2 (5.9)	13 (8.0)	1 (2.9)	16 (10.5)	1 (5.3)	8 (14.5)
Chronic renal diseases	4 (11.8)	10 (6.1)	2 (5.9)	13 (8.5)	1 (5.3)	3 (5.5)
Chronic liver diseases	5 (14.7)	16 (9.8)	3 (8.8)	9 (5.9)	0 (0)	5 (9.1)
Diabetes mellitus	2 (5.9)	14 (8.6)	2 (5.9)	19 (12.4)	2 (10.5)	8 (14.5)
Cancer and hematological diseases	15 (44.1)	47 (28.8)	10	42 (27.5)	8 (42.1)	17 (30.9)
Stroke and Neuromuscular diseases	2 (5.9)	11 (6.7)	2 (5.9)	8 (5.2)	2 (10.5)	7 (12.7)
Immunosuppressant	6 (17.6)	23 (14.1)	4 (11.8)	19 (12.4)	1 (5.3)	4 (7.3)
Pregnancy	0 (0)	0 (0)	0 (0)	2 (1.3)	0 (0)	2 (3.6)
Postmortum within 30 days after delivery	1 (2.9)	0 (0)	0 (0)	0 (0)	1 (5.3)	0 (0)
Current smoking	8 (23.5)	26 (16.0)	6 (17.6)	28 (18.3)	2 (10.5)	7 (12.7)
Received seasonal or influenza A	0 (0)	0 (0)	0 (0)	0 (0)	0 (0)	0 (0)

(H1N1) vaccination						
**Symptoms and Lab findings**						
Fever (temp >=38)	32 (94.1)	147 (90.2)	28 (82.4)	141 (92.2)	19 (100.0)	49 (89.1)
Cough	24 (70.6)	133 (81.6)	**23 (67.6)**	**144 (94.1)****	14 (73.7)	50 (90.9)
Dyspnea	1 (2.9)	15 (9.2)	5 (14.7)	35 (22.9)	0 (0)	11 (20.0)
Hemoptysis	2 (5.9)	4 (2.5)	0 (0)	12 (7.8)	0 (0)	1 (1.8)
CNS symptom	0 (0)	2 (1.2)	4 (11.8	5 (3.3)	0 (0)	2 (3.6)
WBC<4	8 (23.5)	45 (27.6)	**6 (17.6)**	**39 (25.5)***	5 (26.3)	17 (30.9)
L%<20%	22 (64.7)	76 (46.6)	20 (58.8)	98 (64.10	11 (57.9)	32 (58.2)
Platelet<100	11 (32.4)	31 (19.0)	4 (11.8)	23 (15.0)	1 (5.3)	12 (21.8)
ALT >40	**13 (38.2)**	**33 (21.0)***	9 (27.3)	54 (36.2)	3 (15.8)	10 (18.5)
AST >40	12 (35.3)	58 (36.9)	11 (34.4)	65 (43.6)	3 3 (15.8)	14 (26.4)
LDH >300	8 (34.8)	58 (41.1)	**6 (24.0)**	**74 (54.4)****	1 (5.6)	9 (20.0)
CK>200	2 (8.7)	21 (15.1)	5 (20.0)	30 (21.9)	1 (5.6)	9 (20.0)
CRP>8	24 (80.0)	94 (64.8)	**19 (67.9)**	**120 (85.7)***	17 (89.5)	43 (82.7)
ESR >20	8 (44.4)	53 (62.4)	**5 (33.3)**	**74 (71.2)****	2 (33.3)	23 (60.5)
**Outcome**						
In-hospital fatality	1 (2.9)	10 (6.1)	0 (0)	9 (5.9)	0 (0)	1 (1.8)

The boldface values indicate (*P <.05; ** P <.01). Comparison of antiviral therapy <=48 hours from onset with control group (antiviral therapy >48hours from onset).

WBC, white cell ($\times 10^9$/L); L%, lymphocyte percent; PLT, platelet ($\times 10^9$/L); ALT, alanine aminotransferase (U/L); AST, aspartate aminotransferase (U/L); LDH, lactate dehydrogenase (U/L); CK, creatine kinase (U/L); CRP, C-reactive protein (mg/L); ESR, erythrocyte sedimentation rate (mm/h).
