# Supplementary material for: Clinical characteristics and outcomes during a severe influenza season in China during 2017–2018
Source: BMC Infect Dis. 2019 Jul 29;19:668. doi: 10.1186/s12879-019-4181-2 (PMC6664535; doi:10.1186/s12879-019-4181-2)
Supplement: Supplementary file 6 — Table S3. Poisson regression for predicting death of patients during the 2011–2017 and 2017–2018 influenza seasons. (PDF 44 kb) [file 12879_2019_4181_MOESM6_ESM.pdf]

Additional Table 3 **Poisson regression for predicting death of patients during the 2011-2017 and 2017-2018 influenza seasons**

Variables	B	95%CI-Low	95%CI-Up	Wald chi square	P value
**Intercept**	-6.156	-7.472	-4.841	84.104	0
**2017-2018**	**1.112**	**0.373**	**1.852**	**8.687**	**0.003**
**2011-2017 (Ref)**	0[a]				
**WBC>4**	0.089	-0.471	0.649	0.097	0.755
**LDH>300**	0.754	-0.536	2.044	1.313	0.252
**AKI**	-0.504	-1.744	0.736	0.634	0.426
**DIC***	**2.153**	**1.1**	**3.206**	**16.049**	**0.00006**
**Secondary bacterial infection**	0.169	-1.056	1.394	0.073	0.787
**ARDS***	**1.418**	**0.263**	**2.572**	**5.793**	**0.016**
**Respiratory Failure**	0.332	-0.967	1.631	0.251	0.617
**Shock**	-0.93	-2.047	0.188	2.656	0.103
**MOF**	0.212	-1.279	1.703	0.078	0.781
**Glucocorticoids**	1	-0.527	2.527	1.647	0.199
